# Recent advances in the understanding of bronchiolitis in adults

**DOI:** 10.12688/f1000research.21778.1

**Published:** 2020-06-08

**Authors:** Jay H Ryu, Natalya Azadeh, Bilal Samhouri, Eunhee Yi

**Affiliations:** 1 Division of Pulmonary and Critical Care Medicine, Mayo Clinic in Rochester, Rochester, MN, USA; 2 Division of Pulmonary and Critical Care Medicine, Mayo Clinic, Scottsdale, AZ, USA; 3 Division of Anatomic Pathology, Mayo Clinic in Rochester, Rochester, MN, USA

**Keywords:** Aspiration, bronchiole, bronchiolitis, diacetyl, DIPNECH, inhalational injury, small airways, smoking, vaping, virus

## Abstract

Bronchiolitis is injury to the bronchioles (small airways with a diameter of 2 mm or less) resulting in inflammation and/or fibrosis. Bronchioles can be involved in pathologic processes that involve predominantly the lung parenchyma or large airways, but, in some diseases, bronchioles are the main site of injury (“primary bronchiolitis”). Acute bronchiolitis caused by viruses is responsible for most cases of bronchiolitis in infants and children. In adults, however, there is a wide spectrum of bronchiolar disorders and most are chronic. Many forms of bronchiolitis have been described in the literature, and the terminology in this regard remains confusing. In clinical practice, a classification scheme based on the underlying histopathologic pattern (correlates with presenting radiologic abnormalities) facilitates the recognition of bronchiolitis and the search for the inciting cause of the lung injury. Respiratory bronchiolitis is the most common form of bronchiolitis in adults and is usually related to cigarette smoking. Currently, the diagnosis of respiratory bronchiolitis is generally achieved based on the clinical context (smoking history) and chest CT findings. Constrictive (obliterative) bronchiolitis is associated with airflow obstruction and is seen in various clinical contexts including environmental/occupational inhalation exposures, transplant recipients (bronchiolitis obliterans syndrome), and many others. Diffuse idiopathic pulmonary neuroendocrine cell hyperplasia (DIPNECH) is increasingly recognized and can be associated with progressive airflow obstruction related to constrictive bronchiolitis (“DIPNECH syndrome”). Diffuse aspiration bronchiolitis is a form of aspiration-related lung disease that is often unsuspected and confused for interstitial lung disease. Novel forms of bronchiolitis have been described, including lymphocytic bronchiolitis and alveolar ductitis with emphysema recently described in employees at a manufacturing facility for industrial machines. Bronchiolitis is also a component of vaping-related lung injury encountered in the recent outbreak.

## Introduction

“Bronchiolitis” is a term used to designate injury to the bronchioles (small airways with a diameter of 2 mm or less) resulting in inflammation and/or fibrosis ^1^. Many different forms of bronchiolitis have been described over the years, and there is no consensus in classifying these subtypes. Bronchiolitis described in infants and children is a form of acute bronchiolitis representing an infectious process, commonly resulting in respiratory distress and wheezing ^2^. It is caused by a viral infection, most commonly respiratory syncytial virus. In adults, however, a heterogeneous spectrum of bronchiolar disorders is encountered and is more often caused by chronic disease processes ^1, 3– 6^. Etiology, clinical features, radiologic findings, treatment strategies, and prognostic implications vary among these disorders. Thus, it is crucial to distinguish the subtypes of bronchiolitis to optimize management and outcomes.

In this review, current concepts regarding bronchiolitis in adults with an emphasis on recent advances will be described. The forms of bronchiolitis described herein should not be confused with “bronchiolitis obliterans organizing pneumonia” or BOOP (the currently preferred term is “organizing pneumonia” [OP]), which manifests as parenchymal lung disease rather than strictly bronchiolar disease.

## Classification of bronchiolitis

In approaching a heterogeneous entity such as bronchiolitis in adults, a classification scheme is useful in organizing our concepts and facilitating our understanding. Such classification needs to be based on some discriminatory parameter ^3, 7, 8^. It may seem rational to classify bronchiolitis by etiology. However, the underlying cause is often not apparent on clinical presentation. In clinical practice, the task is to recognize the presenting respiratory illness as a form of bronchiolitis, then to identify the underlying cause.

Perhaps the most useful framework in the clinical approach to patients with suspected bronchiolitis is a classification based on underlying histopathologic patterns ( Table 1), which, in large measure, correlate with the clinical and radiologic presentation and help narrow the list of potential causes to be considered ^3^. Ultimately, the treatment of bronchiolitis should be aimed at the cause (e.g. cessation of exposure to offending inhalant) of the disease whenever possible, although it is not always identifiable.

**Table 1.** Classification of bronchiolitis.

Classification	Histopathologic pattern
Primary bronchiolitis	Respiratory bronchiolitis Acute bronchiolitis Constrictive (obliterative) bronchiolitis Follicular bronchiolitis Diffuse aspiration bronchiolitis Diffuse panbronchiolitis Mineral dust airway disease Miscellaneous forms
Bronchiolitis in interstitial lung diseases	Histopathologic features vary with the underlying interstitial lung disease
Bronchiolitis in large airway diseases	Histopathologic features vary with the underlying large airway disease

It should be noted that bronchiolitis can be encountered histopathologically or radiologically as a component of interstitial lung diseases (involving predominantly the lung parenchyma), e.g. hypersensitivity pneumonitis, and large airway diseases such as bronchiectasis ^1^. This issue is exemplified by a disease entity formerly called BOOP, which was characterized by the histologic presence of organizing connective tissue in lumens of small airways, alveolar ducts, and alveoli ^9^. It is generally associated with clinico-radiologic features of parenchymal lung disease including restrictive pulmonary impairment and parenchymal opacities. Thus, this histopathologic entity was later renamed simply “organizing pneumonia” and represents a non-specific pattern of lung injury seen in many diverse clinical contexts, including infectious and non-infectious processes ^1, 10^. In the absence of an identifiable cause, it is referred to as “cryptogenic OP” (COP) and currently classified as a form of idiopathic interstitial pneumonia ^11^. This review, however, focuses on respiratory diseases that manifest predominantly in the bronchioles (“primary bronchiolitis”).

## Respiratory bronchiolitis

Respiratory bronchiolitis (RB) is likely the most common form of bronchiolitis and is usually related to cigarette smoking ^12^. It is characterized by the histologic presence of tan-pigmented macrophages in the respiratory bronchioles ^11^. The presence of RB may not be associated with respiratory symptoms. Radiologically, RB manifests with centrilobular micronodules, which is often how the presence of this disease process is identified ^4, 7, 13^.

Histopathologic lesion of RB is commonly seen in the lung specimens from smokers who are diagnosed with other smoking-related pulmonary diseases including bronchogenic carcinoma and pulmonary Langerhans’ cell histiocytosis, a form of smoking-related interstitial lung disease ^14, 15^. When RB is associated with evidence of interstitial lung disease including diffuse pulmonary infiltrates (typically patchy ground-glass opacities on high-resolution chest CT scan) and pulmonary function impairment, the disease process is referred to as RB-associated interstitial lung disease (RB-ILD) ^16, 17^.

In recent years, RB has been diagnosed on the basis of clinical context (smoking history) and typical chest CT findings (centrilobular ground-glass nodules) without histopathologic confirmation when other potential explanations for the presenting clinico-radiologic features are absent ^7, 13^. Often, these patients have no respiratory symptoms other than a smoker’s cough.

The management focuses on smoking cessation, since RB is related to cigarette smoking in most cases ^13, 17^. Continued smoking may lead some patients to develop evidence of interstitial lung disease including RB-ILD.

In the current era of CT screening for lung cancer, there has been increasing interest in interstitial lung abnormalities (ILAs) encountered in up to 10% of asymptomatic study participants ^18^. The presence of ILAs is associated with increasing age and smoking history ^18, 19^. Diffuse centrilobular nodularity, as can be seen in RB, is a component of these ILAs and is distinguished from “fibrotic” ILAs (reticulation, traction bronchiectasis, and honeycombing) ^18, 19^.

## Acute bronchiolitis

Acute bronchiolitis is a histopathologic pattern of injury underlying the illness referred to as “bronchiolitis” in infants and young children ^1, 2, 20^. It usually represents a viral lower respiratory tract infection, most commonly caused by respiratory syncytial virus ^2, 21^. Histopathology of acute bronchiolitis is characterized by intense acute inflammation of bronchioles with epithelial necrosis and sloughing, along with submucosal edema and peribronchiolar infiltration ^1, 22^.

**Figure 1.**
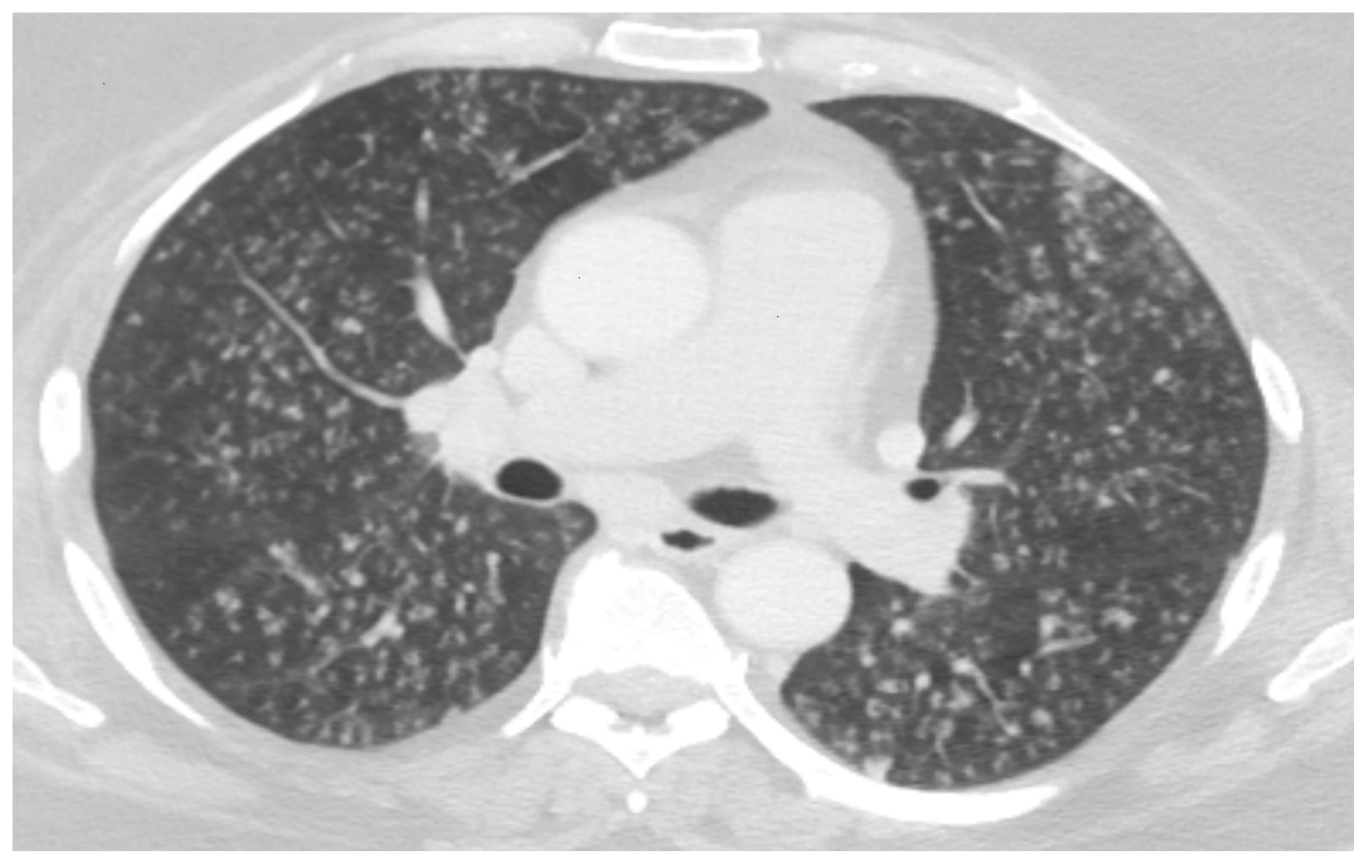
High-resolution chest CT image of a 57-year-old woman with acute bronchiolitis caused by respiratory syncytial virus. Numerous centrilobular nodules are present in both lungs. *This clinical image was obtained during routine clinical practice by the authors. Written informed consent was obtained from the patient for the use and publication of this clinical image.*

In adults, viral respiratory tract infections usually present as tracheobronchitis or pneumonia. However, some adults may experience acute bronchiolitis as the dominant form of viral illness. Various viruses have been associated with acute bronchiolitis in adults and include respiratory syncytial virus, adenovirus, influenza, and parainfluenza ^23– 27^. Non-viral infections including Mycoplasma pneumoniae, Streptococcus pneumoniae, and Haemophilus influenzae as well as acute toxic inhalational injury can also induce acute bronchiolitis ^23, 26– 28^. On chest CT scanning, these patients manifest centrilobular nodules and multifocal tree-in-bud opacities ( Figure 1) rather than bronchial wall thickening (bronchitis) or consolidative/ground-glass opacities (pneumonia) ^1, 23, 24, 26, 27^. The management of acute bronchiolitis in adults is aimed at the underlying cause along with supportive measures which may include mechanical ventilation in severe cases.

## Constrictive (obliterative) bronchiolitis

Constrictive bronchiolitis, also referred to as obliterative bronchiolitis, has many causes and is encountered in various clinical contexts. For example, it is recognized as a form of airway disease associated with autoimmune diseases such as rheumatoid arthritis and Sjögren’s syndrome. Constrictive bronchiolitis underlies “bronchiolitis obliterans syndrome” (BOS; see below), a common form of chronic lung allograft rejection presenting as obstructive lung disease. Constrictive bronchiolitis can also be seen as a late sequela of viral lower respiratory tract infections (Swyer–James–MacLeod syndrome) or toxic inhalational injuries ^1, 29– 33^. Other causes of constrictive bronchiolitis include drugs, inflammatory bowel disease, and paraneoplastic pemphigus ^1, 34, 35^. Constrictive bronchiolitis is a rare form of drug-induced lung disease, but the list of drugs implicated in causing this form of lung injury has been expanding in recent years and include penicillamine, gold, 5-fluorouracil, crack cocaine, afatinib, mesalamine, rituximab, and immune checkpoint inhibitors ^1, 36– 40^. In the absence of an identifiable cause, the term “cryptogenic constrictive bronchiolitis” is used ^1, 31^.

Histologically, constrictive bronchiolitis is characterized by bronchiolar inflammation and peribronchiolar fibrosis that encroaches on the bronchiolar lumen. This constrictive process may result in complete obliteration of the airway lumen ^1, 6^.

Bronchiolitis obliterans refers to a form of constrictive bronchiolitis seen in transplant recipients, predominantly lung or hematopoietic cell transplant ^34, 41– 43^. BOS in lung transplant recipients is defined as a persistent decrease in forced expiratory volume in one second (FEV1) attributable to chronic lung allograft dysfunction and not caused by other identifiable causes ^44^. In BOS, pulmonary function impairment manifests evidence of airflow obstruction and needs to be distinguished from “restrictive allograft syndrome”, which is a restrictive phenotype of chronic lung allograft dysfunction as recently proposed in a consensus statement by the International Society of Heart and Lung Transplantation ^45^.

**Figure 2.**
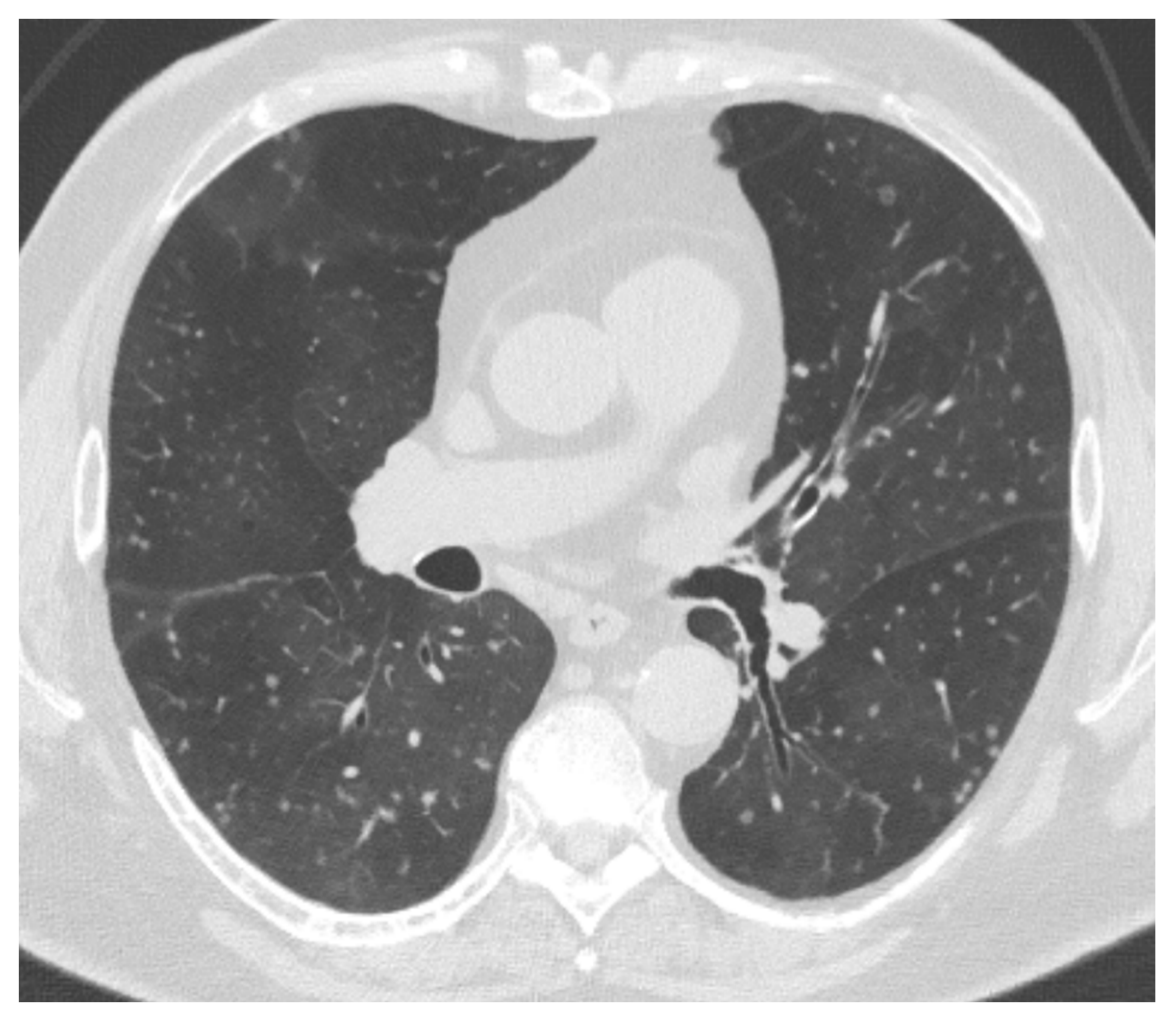
High-resolution chest CT image of a 68-year-old woman, non-smoker, with diffuse idiopathic pulmonary neuroendocrine cell hyperplasia (DIPNECH) syndrome. Multiple pulmonary small nodules are present in the background of mosaic pattern due to patchy air trapping. She had moderate airflow obstruction on pulmonary function testing with a forced expiratory volume in 1 second (FEV _1_) of 55% predicted. *This clinical image was obtained during routine clinical practice by the authors. Written informed consent was obtained from the patient for the use and publication of this clinical image.*

There have been increasing reports in recent years regarding diffuse idiopathic pulmonary neuroendocrine cell hyperplasia (DIPNECH). DIPNECH is an under-recognized respiratory disease characterized by the proliferation of neuroendocrine cells in the airway walls ^46, 47^. It is encountered predominantly in middle-aged women. Radiologically, it is characterized by mosaic attenuation with multiple small nodules ( Figure 2), a combination that is highly suggestive of this disease ^48^. DIPNECH has been classified as a preinvasive lesion in the category of neuroendocrine tumors in the 2015 World Health Organization (WHO) classification of lung tumors ^49^.

Some patients with DIPNECH may be asymptomatic and identified on the basis of abnormal chest CT findings, whereas the clinical course in other patients is characterized by the development of carcinoid tumors or progressive airflow obstruction. The latter situation represents constrictive bronchiolitis associated with DIPNECH (termed “DIPNECH syndrome”) and is thought to be mediated by peptides secreted by hyperplastic neuroendocrine cells leading to peribronchiolar fibrosis and progressive narrowing of the small airways ^46, 47, 50^. A definitive diagnosis requires histopathologic confirmation, usually surgical lung biopsy. For patients with DIPNECH syndrome and progressive obstructive lung impairment, various therapeutic options have been tried including oral and inhaled glucocorticoids, cytotoxic agents, and somatostatin analogues, but an optimal/effective treatment has not been identified ^46, 47, 51^. A recent report described three patients with DIPNECH syndrome who experienced improvement with sirolimus (an inhibitor of mechanistic target of rapamycin [mTOR] pathway) therapy ^52^. In most patients, DIPNECH is associated with an indolent course, but progressive respiratory insufficiency necessitating lung transplantation has been reported. Pulmonary neuroendocrine tumorlets (neuroendocrine cell hyperplasia less than 5 mm in size) are commonly seen in patients with DIPNECH who are also at risk for carcinoid tumors ^46, 53^.

In recent years, several inhalational causes of constrictive bronchiolitis have been described. These have included inhalational exposures associated with military service in Southwest Asia and Afghanistan and the use of flavoring chemicals in the food industry ^28, 54, 55^. Diacetyl was identified as the chemical responsible for constrictive bronchiolitis occurring in workers in microwave popcorn manufacturing ^56^. Similar respiratory illnesses associated with exposure to diacetyl and 2,3-pentanediol have been identified in workers in other food production settings, including cookie production and coffee-processing facilities ^28, 57^. It seems likely that other environmental and occupational exposures will be implicated in the development of constrictive bronchiolitis and account for some non-smokers diagnosed with chronic obstructive pulmonary disease (COPD) ^58, 59^.

## Follicular bronchiolitis

Follicular bronchiolitis is characterized histologically by non-neoplastic lymphoid hyperplasia of the bronchus-associated lymphoid tissue ^1, 6^. Imaging features on high-resolution CT of the chest are bilateral presence of small centrilobular nodules and patchy ground-glass opacities ^1, 4, 60^. Follicular bronchiolitis is usually encountered in patients with connective tissue diseases (e.g. rheumatoid arthritis and Sjögren syndrome) and immunodeficiency disorders ^1, 60, 61^. In some cases, an underlying cause may not be identifiable.

Recently, a novel familial form of autoimmune disorder associated with follicular bronchiolitis was described. COPA syndrome is a rare disease caused by heterozygous missense mutations in the gene encoding coatomer subunit alpha (COPA) ^62– 64^. This monogenic disorder is inherited in an autosomal-dominant manner with variable expressivity. The coatomer protein complex plays a role in intracellular vesicle trafficking, and impaired protein transport is thought to result in abnormal cellular autophagy and immune dysregulation ^62, 63^. Affected family members manifest arthritis, circulating autoimmune antibodies (particularly rheumatoid factor), follicular bronchiolitis, and recurrent respiratory infections ^64– 66^. Diffuse alveolar hemorrhage may occur in about one-half of patients ^65^. Chest CT scan demonstrates diffuse small lung nodules often associated with small cysts ^64, 66^. These patients tend to experience gradual progression of their lung disease despite treatment with glucocorticoids and other immunomodulator therapy; some have undergone lung transplantation ^65^.

## Diffuse aspiration bronchiolitis

Diffuse aspiration bronchiolitis is a form of aspiration-related lung disease resulting from chronic recurrent aspiration, which is often occult ^67– 72^. This disease presents with an insidious onset of cough that persists, sometimes accompanied by exertional dyspnea, and bilateral pulmonary infiltrates that may be mistaken for interstitial lung disease. Although risk factors for aspiration such as gastroesophageal reflux disease and sedative medication use are commonly present in these patients, the diagnosis is frequently unsuspected until foreign bodies representing food particles are identified on lung biopsy ^67, 70– 72^.

In contrast to other more recognized forms of aspiration-related lung diseases such as aspiration pneumonia and aspiration pneumonitis, radiologic findings associated with diffuse aspiration bronchiolitis on CT consist of centrilobular nodules and tree-in-bud opacities ^67, 68, 71, 73^.

The management of diffuse aspiration bronchiolitis is aimed at the underlying risk factors for aspiration such as gastroesophageal reflux disease, the use of sedating medications, and neuromuscular disorders ^72^.

## Miscellaneous forms of bronchiolitis

Although the classification scheme depicted in Table 1 includes most forms of bronchiolitis encountered in clinical practice, new forms of bronchiolitis continue to be discovered. A unique histopathologic pattern of lymphocytic bronchiolitis and alveolar ductitis with emphysema was recently described in five never-smokers employed at a manufacturing facility for industrial machines ^74^. These patients manifested evidence of airflow obstruction, impaired gas exchange, and centrilobular emphysema. No cause has yet been identified for this previously unrecognized occupational lung disease.

Recently, there has been a multistate outbreak of respiratory illnesses associated with the use of electronic cigarettes (e-cigarettes) or vaping ^75^. The exact chemical or chemicals responsible for this illness have not yet been identified. Lung biopsy obtained in these patients has revealed patterns of acute lung injury including diffuse alveolar damage or fibrinous OP with a bronchiolocentric distribution and accompanied by bronchiolitis ^76^. RB-ILD and acute eosinophilic pneumonia have also been reported to occur with e-cigarette use ^77– 79^.

## Summary

Bronchiolitis is encountered commonly, sometimes as a relatively minor component of the disease process that mainly affects the lung parenchyma or large airways but at other times may be the principal site of the lung injury (primary bronchiolitis). In adults, the spectrum of bronchiolar injury is broad in terms of the histopathologic pattern and causes. Accordingly, imaging features on chest CT as well as pulmonary function findings will vary among different forms of bronchiolitis. Correlation of the clinical context with CT and pulmonary function findings may enable a working diagnosis in some patients, while lung biopsy may be needed for diagnostic clarification in others. Identification of the type of bronchiolitis and the underlying cause, whenever possible, optimizes management and outcomes.
